# Supplementary material for: Dandelion pappus morphing is actuated by radially patterned material swelling
Source: Nat Commun. 2022 May 6;13:2498. doi: 10.1038/s41467-022-30245-3 (PMC9076835; doi:10.1038/s41467-022-30245-3)
Supplement: Supplementary file 1 — Supplementary Information [file 41467_2022_30245_MOESM1_ESM.pdf]

Dandelion pappus morphing is actuated by radially patterned material swelling
Supplementary Information

Supplementary Figures

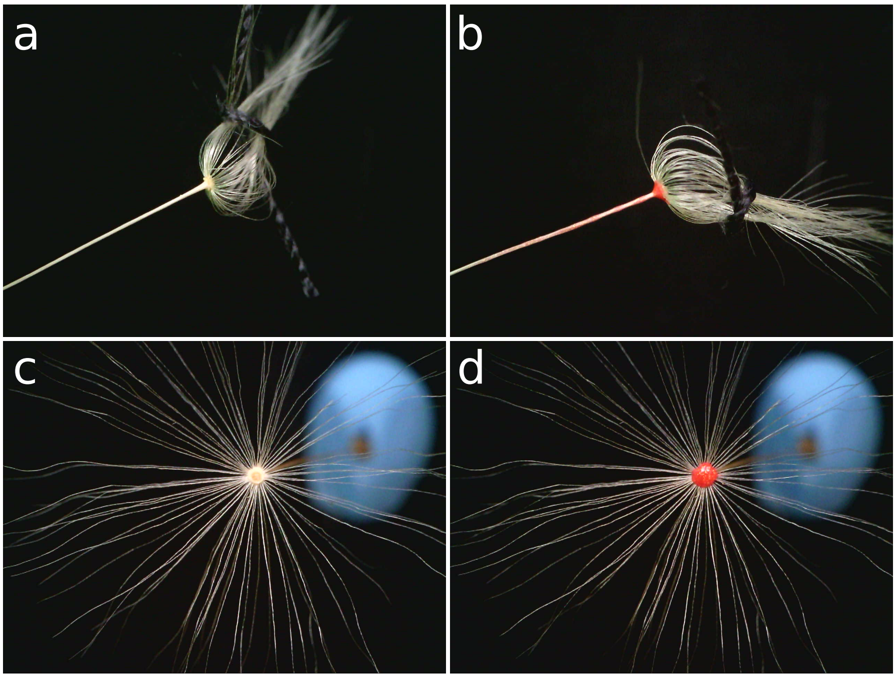

Supplementary Figure 1: Images of the pappus illustrating methods of apical plate blocking. **a** tied pappus hairs before applying resin to lower side, **b** pappus after applying resin to lower side, **c** pappus before applying resin to upper side, **d** pappus after applying resin to upper side.

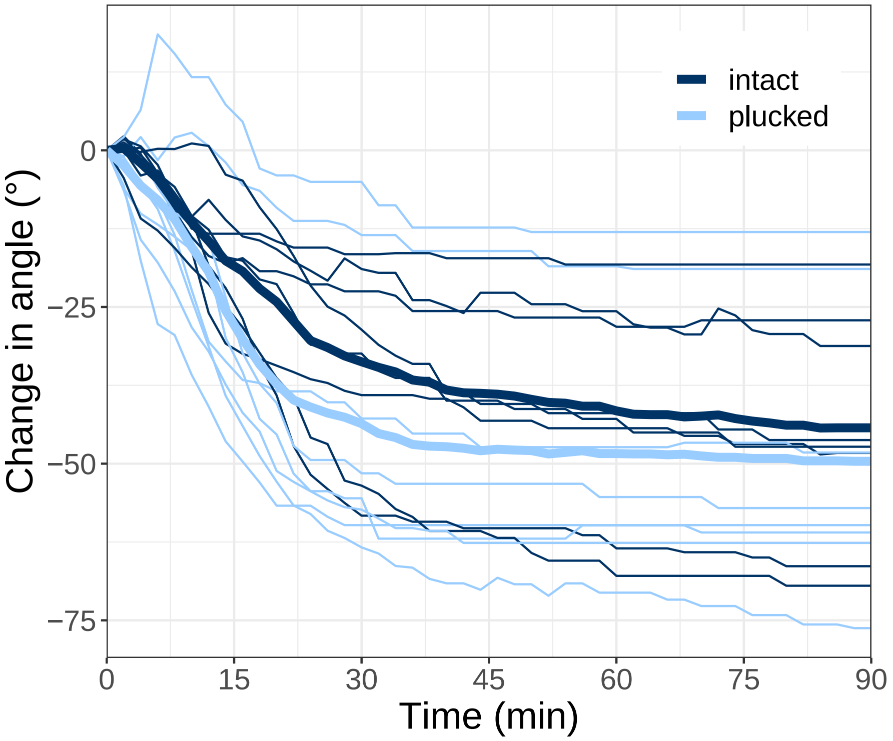

Supplementary Figure 2: Quantification of pappus angle (the angle between outermost filaments) for plucked and intact dandelion pappi. Each sample is represented by a separate line with thicker lines indicating the mean. Dark blue indicates intact samples and light blue indicates plucked samples. $n = 8$ biologically independent samples per treatment.

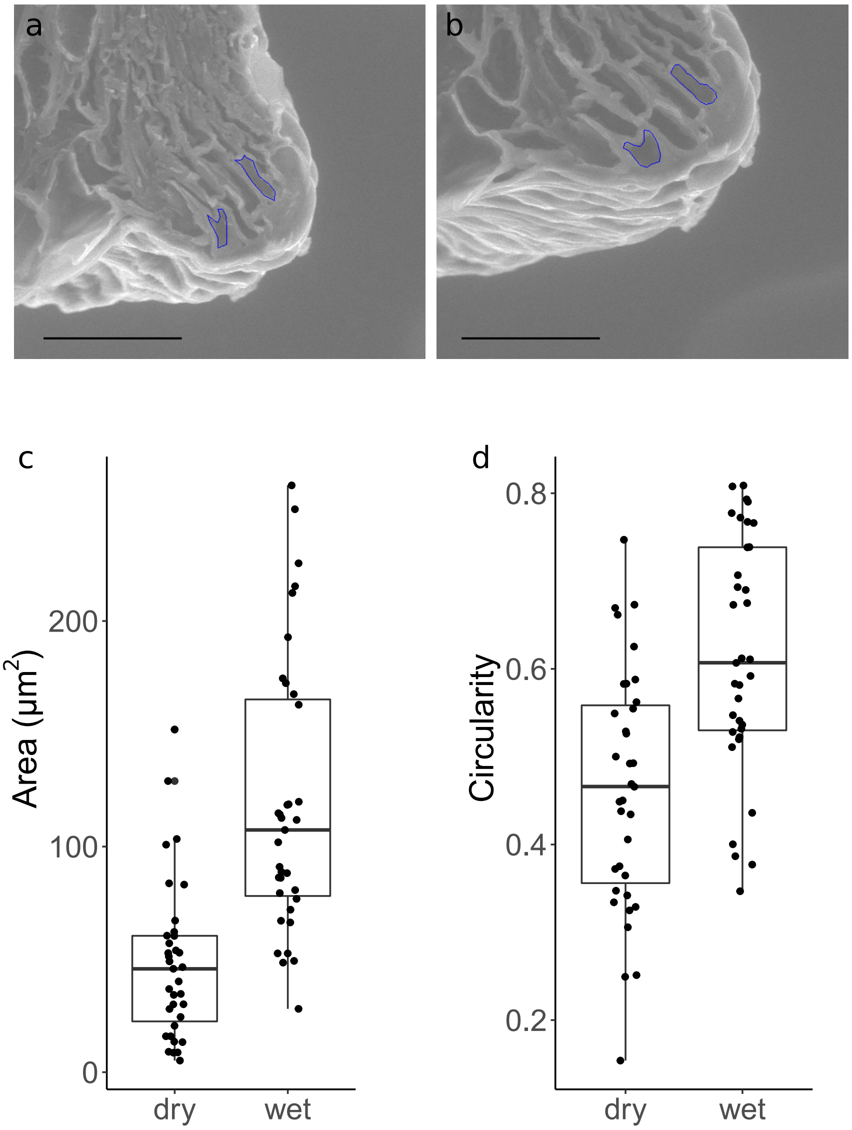

Supplementary Figure 3: Cells in the cortex expand and change shape. **a**-**b** ESEM images of part of the cortex tissue with outlines of two cells marked in the dry **a**, and wet **b** states. Scale bars are 50 μm and images are representative of $n = 9$ biologically independent samples. Cell area **c** and circularity **d** of cortex cells in dry and wet states determined from ESEM images, $n = 35$ cells from 9 biologically independent samples. For boxplots, the centre line shows the the median, hinges show the first and third quartile, and whiskers extend to the largest value no further than 1.5 times the interquartile range.

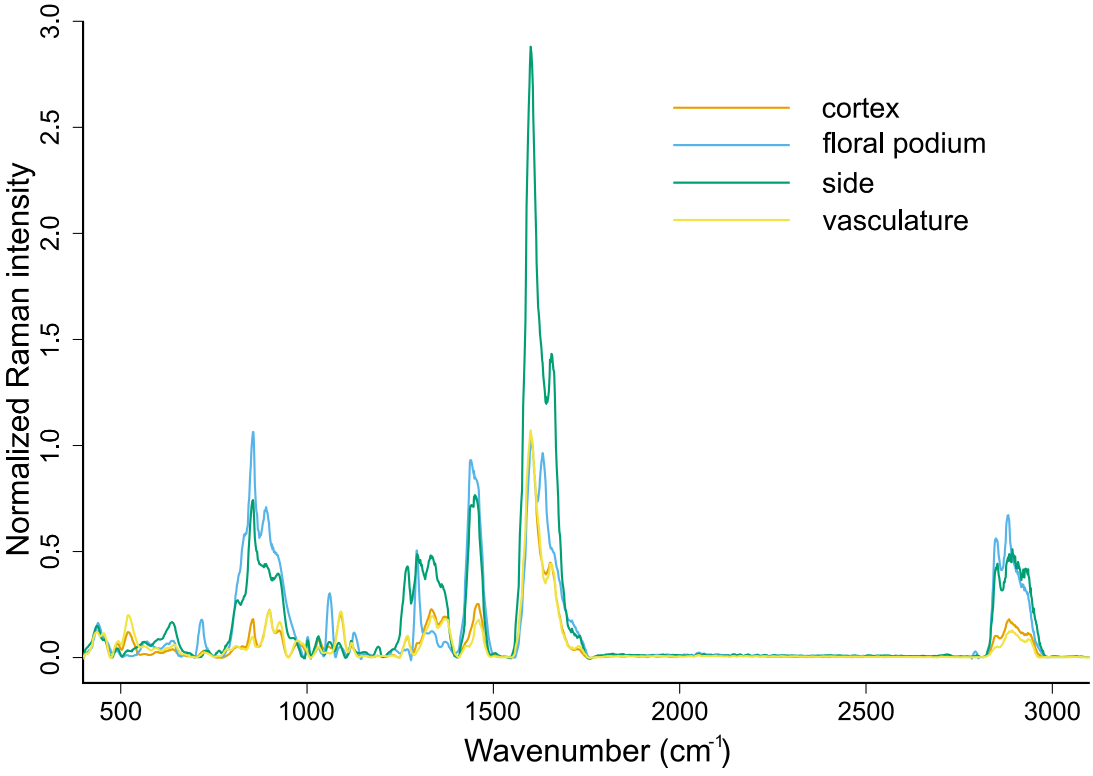

Supplementary Figure 4: Average Raman spectra for different apical plate domains. Data are normalized to a peak at 321 cm^{-1} arising from the CaF_2 substrate, $n = 5$ biologically independent samples.

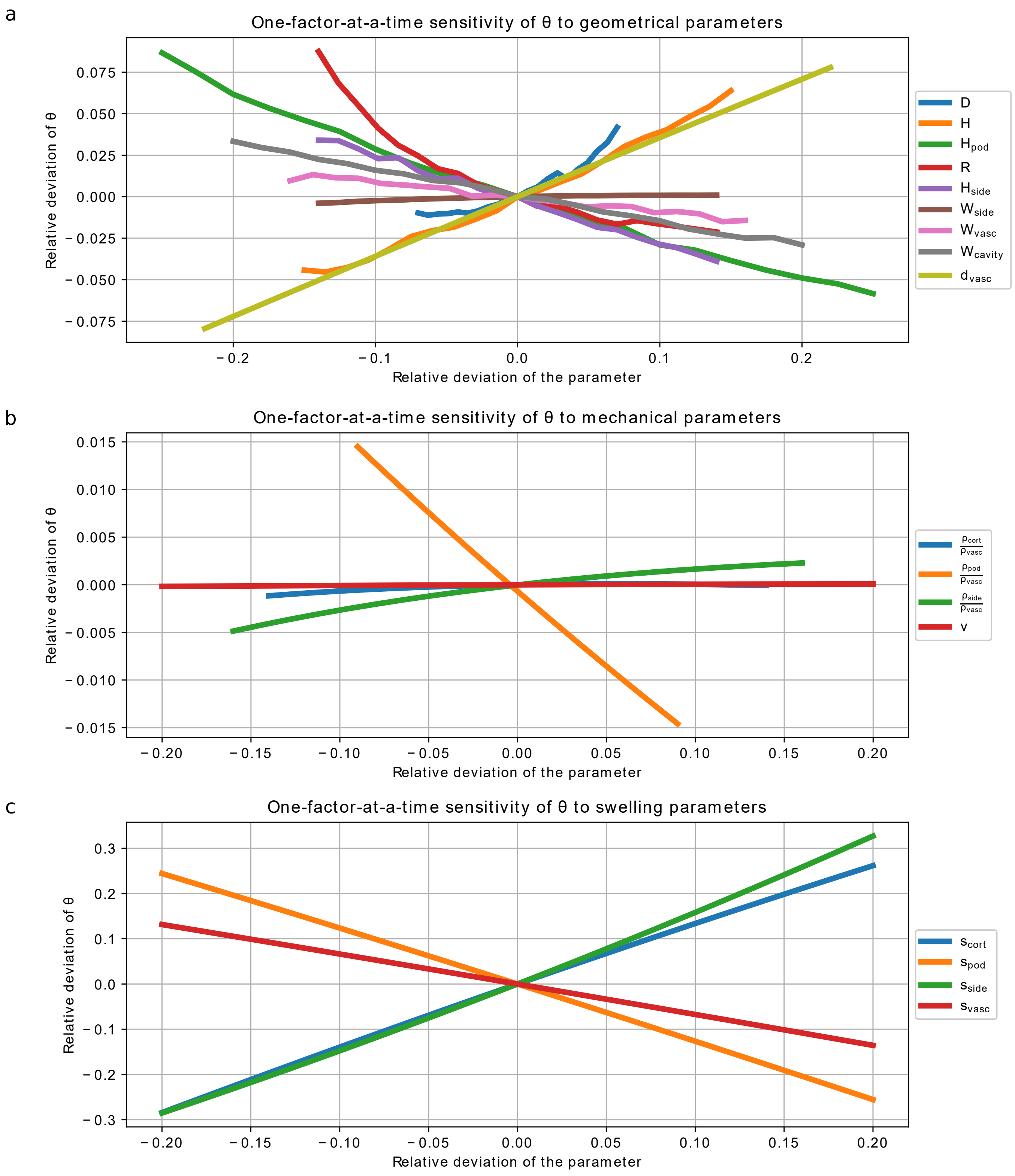

Supplementary Figure 5: One-factor-at-a-time sensitivity analysis across the range of parameter variations. **a** Sensitivity analysis of geometrical parameters. **b** Sensitivity analysis of mechanical parameters. **c** Sensitivity analysis of swelling parameters.

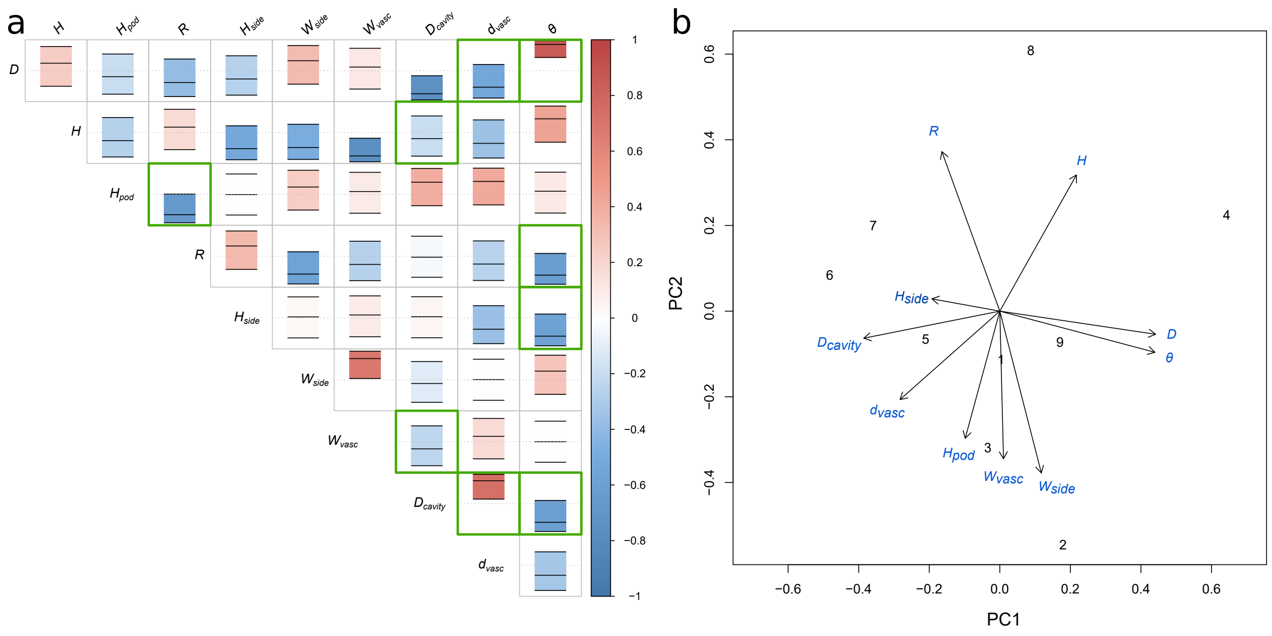

Supplementary Figure 6: Relationship between observed geometrical features. **a** Pearson's correlation matrix illustrating the relationship between observed geometrical parameters. The colour scale and central bar of each box indicates the correlation coefficient, outer bars give the 95% confidence interval limits. Green boxes indicate a statistically significant result at $p < 0.1$. **b** biplot of the first two principal components from a principal component analysis of geometrical parameters. $n = 9$ biologically independent samples.

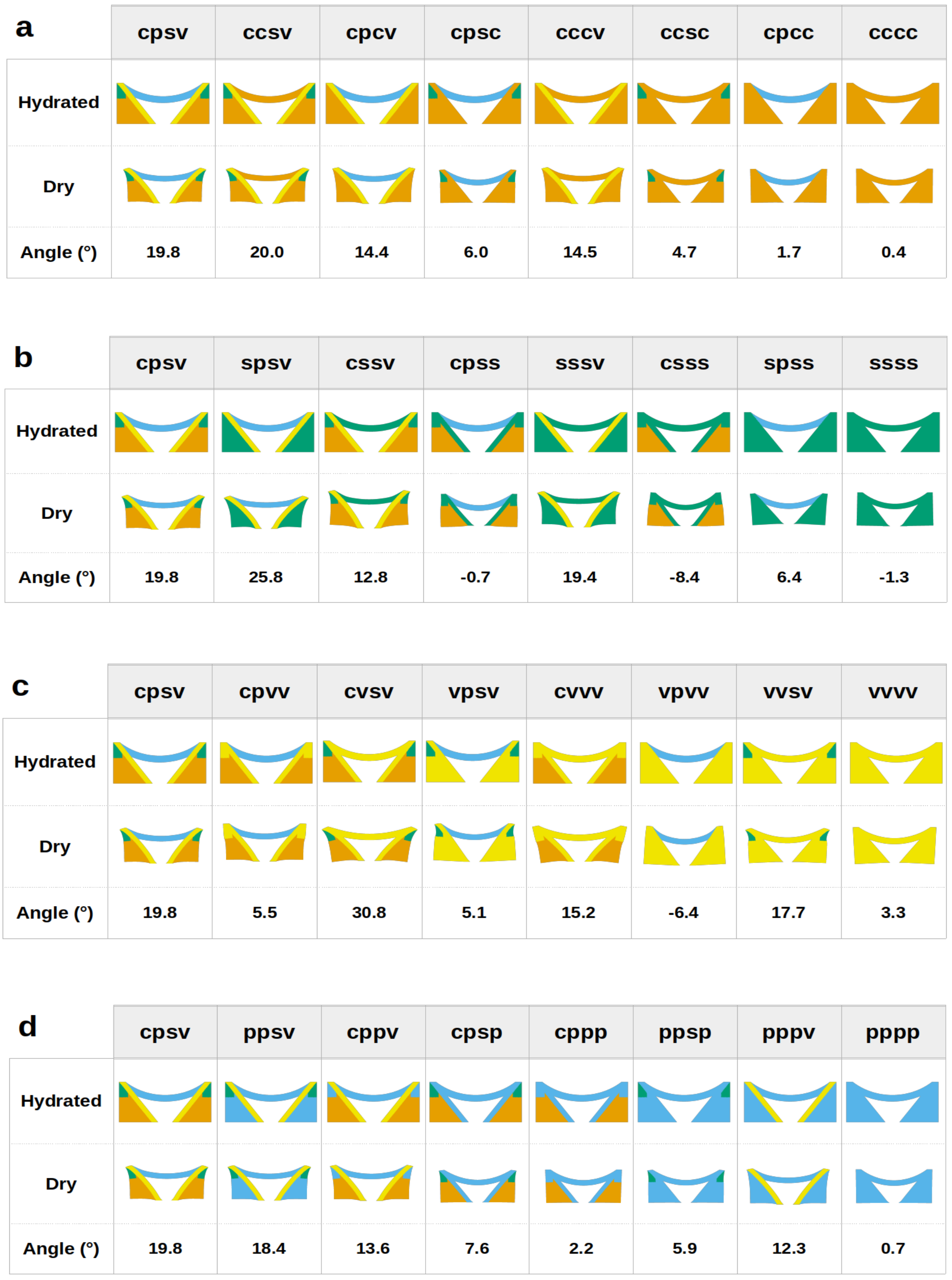

Supplementary Figure 7: In silico mutations. Each region is substituted with alternative tissue types. Tissue types are denoted by letters (c=cortex, p=floral podium, s=side, v=vasculature), and the different geometrical regions are marked by the order of letters in the word designing the mutant (1st letter=cortex region, 2nd letter=floral podium region, 3rd letter=side region, 4th letter=vasculature region in the reference model). This way the reference model is denoted by the word âcpsvâ, and a mutant in which the vasculature region is turned into cortex is denoted âcpscâ. **a** substitutions with cortex-like tissue. **b** substitutions with side-like tissue. **c** substitutions with vasculature-like tissue. **d** substitutions with floral podium-like tissue.

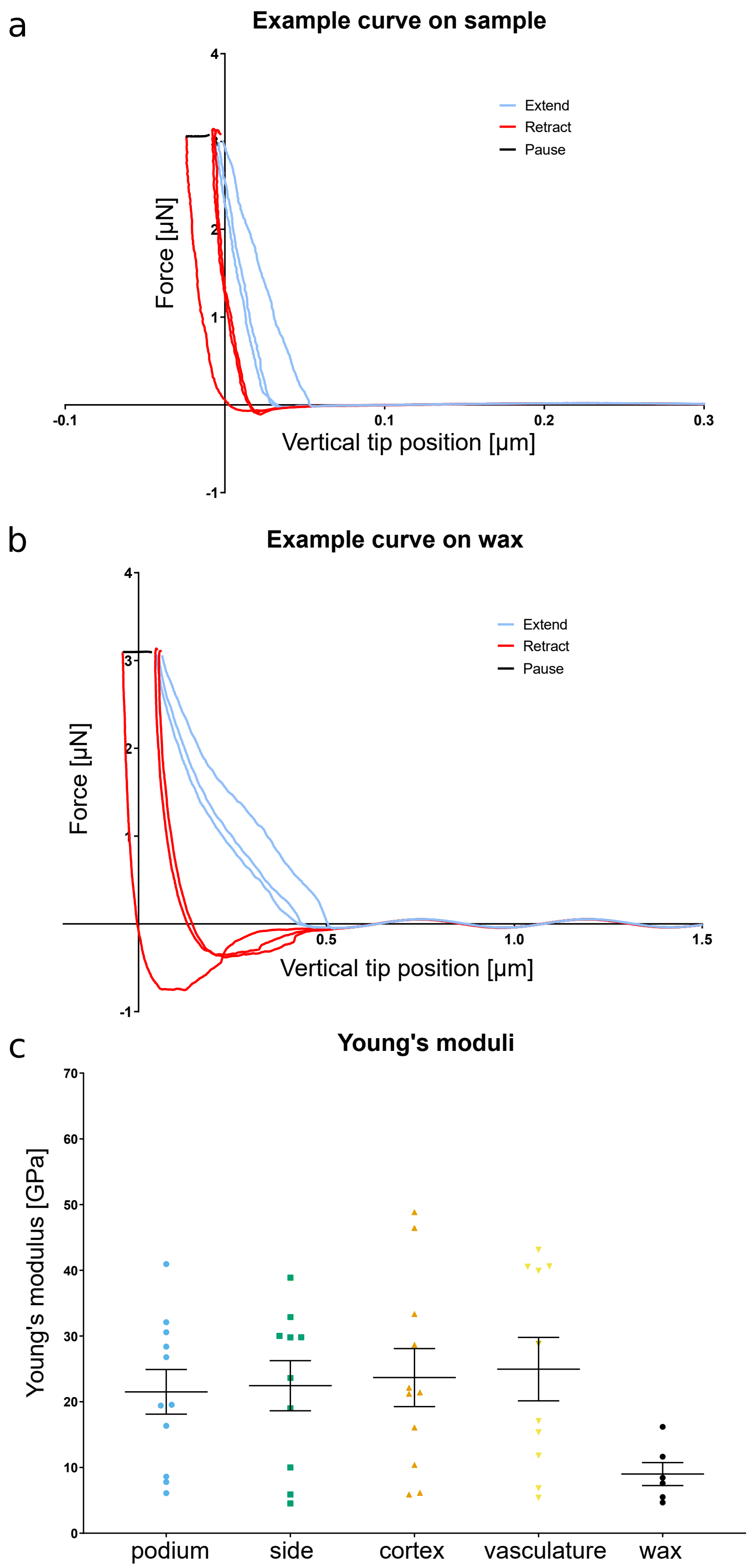

Supplementary Figure 8: Example curves acquired on the sample **a**, and on wax **b**. Curves in light blue are extension segments, retraction segments are represented in red. Effective Youngâs modulus was measured on the last red segment (first from the left). Note that the x scale is not the same on these two graphs. **c** the median of the effective Youngâs modulus for each of the 4 regions of the apical plate (about 30 points for each measurement, numbers of biologically independent samples were $n = 11$ for podium and cortex, $n = 10$ for side and vasculature, $n = 6$ for wax). Vertical bars span the mean plus or minus the standard error of the mean.

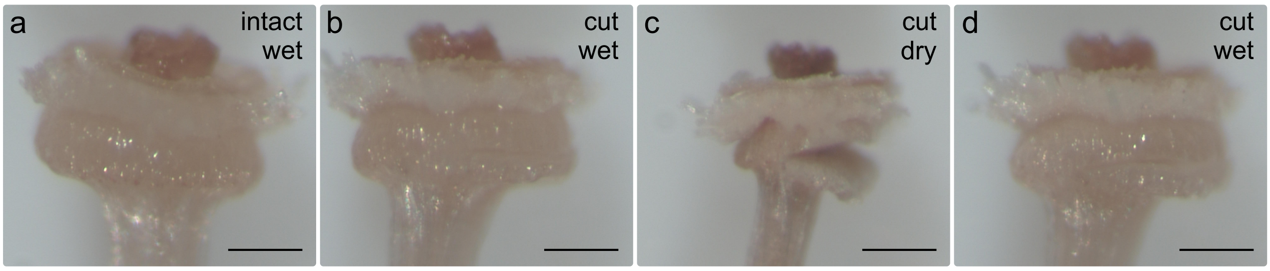

Supplementary Figure 9: Mechanical stress in hydrated and dehydrated samples. Images of a hydrated apical plate sample intact **a**, after an incision was made **b**, after dehydration **c**, and after rehydration **d**. Pappus hairs were removed to allow observation of the structure without interference and the same sample is shown in each panel. Scale bars are 200 μm. This image sequence is representative of $n = 12$ biologically independent samples.

Supplementary Note 1

1 Modelling the hygroscopic actuator

1.1 Formulation

We model the actuator as a two-dimensional, isotropic, linearly elastic material that undergoes shrinkage due to loss of water. Deformations are assumed to be sufficiently small and shrinkage is treated like thermal dilation in elastic solids [3]. A material point with coordinates (x_1, x_2) is displaced to $(x_1 + u_1, x_2 + u_2)$, so that the vector field u is the displacement field. The observed strain, ε is the symmetrized gradient of u,

$$\varepsilon = \frac{1}{2}\left[\nabla u + (\nabla u)^T\right], \tag{1}$$

where ∇u is the gradient of u and $(\nabla u)^T$ stands for the transpose of ∇u. Swelling corresponds to a target isotropic strain

$$\varepsilon_s = sI, \tag{2}$$

with I the identity tensor. Elastic deformations are associated with the difference between observed strain and target strain, so that elastic strain is

$$\varepsilon_{el} = \varepsilon - \varepsilon_s. \tag{3}$$

If the material were isotropic and homogeneous across the whole domain, elastic strain, ε, would vanish and so would stress. We assume the material to be linearly elastic, characterised by a fourth order elastic tensor E, so that stress tensor is given by

$$\sigma = E : \varepsilon_{el}, \tag{4}$$

where $E : \varepsilon$ is the contraction of the two tensors. The force balance reads

$$\nabla \cdot \sigma = 0. \tag{5}$$

At boundaries, we assume either no external force or fixed displacement (see below).

The material is isotropic, therefore the only non-zero components of the elastic tensor are

$$\{E_{1111}, E_{2222}, E_{1122}, E_{1212}\} = \{2\mu + \lambda, 2\mu + \lambda, \lambda, \mu\}, \tag{6}$$

with Lamé's parameters λ and μ linked to the Young's modulus E and Poisson's ratio ν by

$$\mu \;=\; \frac{E}{2(1+\nu)}, \qquad \lambda \;=\; \frac{E\nu}{1-\nu^2}.$$

Note that this formulation simplifies down to the usual linear elasticity formulation in the case where the hygroscopic swelling s vanishes.

1.2 The implementation

We use the finite element method to solve the elastic problem. More specifically we use FreeFem [2] for mesh creation and solving, as well as visualisation. FreeFem requires the variational formulation of the problem. The solution for the displacement u is such that the integral over the domain of the bilinear form $a(u, v) = \sigma : \epsilon$ vanishes for any test function v, with $\epsilon_{ij} = 1/2(\partial_i v_j + \partial_j v_i)$ the virtual strain corresponding to the virtual displacement v. Using the components of the tensors, the bilinear form takes the form (using Einstein notation implying summations over repeated indices),

$$\begin{aligned} a(u,v) &= \sigma_{ij}\epsilon_{ij} \\ &= E_{ijkl}\epsilon_{ij}\epsilon_{kl} - sE_{kkij}\epsilon_{ij}. \end{aligned}$$

The components of the displacement vector fields u and v are implemented as piecewise degree 2 polynomials (P2 elements). The material properties E and s are implemented as piecewise constant fields (P0 elements). The Poisson ratio ν is considered a constant.

Code availability: The implementation of the model as a FreeFem[2] script can be found in the following repository: `https://doi.org/10.5281/zenodo.6424448`

1.3 The input parameters of the model

We defined 4 regions as shown in Figure 5c: cortex, vasculature, floral podium, and side. Each region is characterised by its geometrical parameters, see Figure 5a, by its elastic modulus, and by its swelling parameter.

The total number of model parameters is 17 (see Tables 1 and 2 of the main text), among which 12 are obtained based on experimental data, 1 has a standard value taken from the literature, and 4 are estimated by optimisation.

- **Geometric parameters** (8 parameters): all the lengths that define the geometry of the actuator. Their values are experimental data reported in Table 1.
- **Elastic parameters** (3+1 parameters): a common Poisson ratio ν for all regions, and 3 ratios of region moduli to the vasculature modulus (the absolute values of moduli do not matter because there is no external force applied and so no force scale). The Poisson ratio is taken to be $\nu = 0.29$, typical of woody tissues [4]. Similarily, in order to estimate the ratio of elastic moduli of the regions, we make use of the observation that the Young modulus of a woody plant tissue exhibits a linear to cubic dependence on the relative density, depending on the direction of loading when cellular structure is highly anisotropic [1]. Here we assumed an isotropic material and a linear dependence of Young moduli on the density, and the relative Young's moduli are approximated by relative densities of regions (quantified as the area of cell wall per unit tissue area). The measured density ratios are given in Table 1.
- **Boundary condition** (1 parameter): vasculature displacement d_{vasc} between the dry and wet states, its mean is 15.3 μm.
- **Swelling parameters** (4 parameters): one per region. As these cannot be directly measured, we developed a specific approach to estimate them (see below).

1.4 The reference model

As described above in section 1.3, we measured or chose plausible values for all parameters other than the 4 swelling parameters. We used an optimisation method to determine these 4 unknown parameters. Their values are adjusted so that simulated ratio of dry to wet area of regions $r_i, i \in \{p, s, c, v\}$ (p, s, c, v refer to podium, sides, cortex, and vasculature, respectively) are as close as possible to measured area ratios: means $\overline{R_i}$ and standard deviations σ_{R_i}, with $i \in \{p, s, c, v\}$. Accordingly, we sought the swelling parameters $s_i, i \in \{p, s, c, v\}$ that minimise the function

$$f(\{s_i\}) = \sum_{i\in\{p,s,c,v\}} \frac{(r_i - \overline{R_i})^2}{\sigma^2_{R_i}}. \quad (7)$$

The weights $1/\sigma^2_{R_i}$ allow us to account for the uncertainty in measurements so that robustly measured area changes have the highest weights in the optimisation. We thus obtained the values of swelling parameters shown in Supplementary Table 1.

Supplementary Table 1. Intrinsic swelling parameters per region in the reference model.

Region	intrinsic swelling parameter (s)	intrinsic area change ($A_{hydrated}/A_{dry}$)
floral podium	-0.44	1.65
sides	-0.57	1.97
cortex	-0.46	1.70
vasculature	-0.24	1.28

We performed a series of perturbations of this reference system and predicted the corresponding behaviours, as detailed in the main text.

1.5 Sensitivity analysis and correlation coefficients

To investigate the robustness of the reference model, we characterised the sensitivity of predictions to parameters. Each measured parameter x_i has some experimental variability and is characterised by its mean $\overline{x_i}$, its standard deviation σ_i, and coefficient of variation $V_i = \sigma_i/\overline{x_i}$. The sensitivity ϕ_i of an output quantity a to the value of parameter x_i is defined as

$$\phi_i = \frac{\overline{x_i}}{\overline{a}} \frac{\partial a}{\partial x_i}, \qquad (8)$$

where $\overline{a}$ is the predicted value of a in the reference model (i.e. when all parameters x_i are equal to their measured mean values $\overline{x_i}$). Notice that the sensitivities ϕ_i are normalized, non-dimensional quantities, and thus sensitivities of the output to different input parameters can be compared.

In order to further test the model, we sought to compare these sensitivities to observed natural variability between samples. Such comparison is not directly possible because parameters do not vary independently in actual samples. Indeed the correlation coefficients between observed parameters x_i and x_j,

$$c_{ij} = \frac{\overline{(x_i - \overline{x_i})(y_j - \overline{y_j})}}{\sigma_i \sigma_j}, \qquad (9)$$

are generally non zero. We therefore predicted the coefficient of correlation between model output a conditioned by observed correlations in experiments as follows.

Based on sensitivities, the predicted value of a varies as

$$\frac{a - \overline{a}}{\overline{a}} = \sum_i \phi_i \frac{x_i - \overline{x_i}}{\overline{x_i}}, \qquad (10)$$

for small fluctuations in parameters around their mean values.

In particular, based on equation (10) and provided observed correlation coefficients c_{ij}, the coefficient of variation of a is computed as

$$V_a^2 = \sum_{ij} V_i \phi_i c_{ij} V_j \phi_j, \qquad (11)$$

while the correlation coefficient between the output parameter a and the input parameters x_i is found to be

$$c_{ai} = \sum_j c_{ij} \phi_j \frac{V_j}{V_a}, \qquad (12)$$

which can be directly compared to experimental values.

References

[1] Lorna J. Gibson. The hierarchical structure and mechanics of plant materials. *Journal of The Royal Society Interface*, 9(76):2749–2766, November 2012. Publisher: Royal Society.

[2] F. Hecht. New development in FreeFem++. *J. Numer. Math.*, 20(3-4):251–265, 2012.

[3] L.D. Landau and E.M. Lifshitz. *Theory of Elasticity.* Pergamon, New York, 3rd ed. edition, 1986.

[4] Robert J Ross. Wood handbook: wood as an engineering material. *USDA Forest Service, Forest Products Laboratory, General Technical Report FPL-GTR-190, 2010: 509 p. 1 v.*, 190, 2010.
