# Supplementary material for: Dandelion pappus morphing is actuated by radially patterned material swelling
Source: Nat Commun. 2022 May 6;13:2498. doi: 10.1038/s41467-022-30245-3 (PMC9076835; doi:10.1038/s41467-022-30245-3)
Supplement: Supplementary file 3 — Description of Additional Supplementary Files [file 41467_2022_30245_MOESM3_ESM.pdf]

Description of Additional Supplementary Files

File Name: Supplementary Movie 1
Description: Top-down view of the pappus in the moisture chamber. Moisture was added to the chamber for the full duration of the imaging (1 hour) and images captured every 30 seconds. Scale bar is 2 mm.

File Name: Supplementary Movie 2
Description: ESEM video of apical plate hydration. Chamber pressure is set to 6.3 Torr to stimulate water condensation on the sample. Once submerged with water, the chamber pressure is briefly reduced to 5.5 Torr to cause surface water to evaporate and the sample to become visible again
