# Supplementary material for: Dandelion pappus morphing is actuated by radially patterned material swelling
Source: Nat Commun. 2022 May 6;13:2498. doi: 10.1038/s41467-022-30245-3 (PMC9076835; doi:10.1038/s41467-022-30245-3)
Supplement: Supplementary file 6 — Reporting Summary [file 41467_2022_30245_MOESM6_ESM.pdf]

nature portfolio

Corresponding author(s): Naomi Nakayama and Arezki Boudaoud

Last updated by author(s): Apr 18, 2022

Reporting Summary

Nature Portfolio wishes to improve the reproducibility of the work that we publish. This form provides structure for consistency and transparency in reporting. For further information on Nature Portfolio policies, see our Editorial Policies and the Editorial Policy Checklist.

Statistics

For all statistical analyses, confirm that the following items are present in the figure legend, table legend, main text, or Methods section.

n/a	Confirmed	
☐	☒	The exact sample size (n) for each experimental group/condition, given as a discrete number and unit of measurement
☐	☒	A statement on whether measurements were taken from distinct samples or whether the same sample was measured repeatedly
☐	☒	The statistical test(s) used AND whether they are one- or two-sided<br>*Only common tests should be described solely by name; describe more complex techniques in the Methods section.*
☒	☐	A description of all covariates tested
☐	☒	A description of any assumptions or corrections, such as tests of normality and adjustment for multiple comparisons
☐	☒	A full description of the statistical parameters including central tendency (e.g. means) or other basic estimates (e.g. regression coefficient) AND variation (e.g. standard deviation) or associated estimates of uncertainty (e.g. confidence intervals)
☐	☒	For null hypothesis testing, the test statistic (e.g. F, t, r) with confidence intervals, effect sizes, degrees of freedom and P value noted<br>*Give P values as exact values whenever suitable.*
☒	☐	For Bayesian analysis, information on the choice of priors and Markov chain Monte Carlo settings
☒	☐	For hierarchical and complex designs, identification of the appropriate level for tests and full reporting of outcomes
☐	☒	Estimates of effect sizes (e.g. Cohen's d, Pearson's r), indicating how they were calculated

Our web collection on statistics for biologists contains articles on many of the points above.

Software and code

Policy information about availability of computer code

Data collection	NA
Data analysis	R 4.1.0<br>RStudio 1.4.1717<br>R packages used:<br>deldir<br>raster<br>rgeos<br>scales<br>Chemospec<br>R.utils<br>pracma<br>plyr<br>lsr<br>ggplot2<br>lme4<br>corrplot<br><br>FreeFEM++ v4.9

For manuscripts utilizing custom algorithms or software that are central to the research but not yet described in published literature, software must be made available to editors and reviewers. We strongly encourage code deposition in a community repository (e.g. GitHub). See the Nature Portfolio guidelines for submitting code & software for further information.

Data

Policy information about availability of data

All manuscripts must include a data availability statement. This statement should provide the following information, where applicable:

- Accession codes, unique identifiers, or web links for publicly available datasets
- A description of any restrictions on data availability
- For clinical datasets or third party data, please ensure that the statement adheres to our policy

Data are provided with the manuscript and are available at the following repository: https://doi.org/10.5281/zenodo.6460887

Field-specific reporting

Please select the one below that is the best fit for your research. If you are not sure, read the appropriate sections before making your selection.

☒ Life sciences ☐ Behavioural & social sciences ☐ Ecological, evolutionary & environmental sciences

For a reference copy of the document with all sections, see nature.com/documents/nr-reporting-summary-flat.pdf

Life sciences study design

All studies must disclose on these points even when the disclosure is negative.

Sample size	No sample size calculations were performed. Sample sizes were comparable to other studies in this field (e.g. Burgert et al. (2007) Planta 226:981; Brule et al. (2019) J. R. Soc. Interface, 16:20190454)
Data exclusions	1 sample was excluded from local expansion analysis as vasculature was not clearly visible on both left and right hand sides.
Replication	Experimentation was carried out at different times for each individual and data were reproducible across all samples.
Randomization	Samples were selected at random from each plant.
Blinding	Blinding was not possible data collection as regions had to be identified correctly. Similarly, this was not carried out for image analysis as consistent features had to be identified from one time point to another.

Reporting for specific materials, systems and methods

We require information from authors about some types of materials, experimental systems and methods used in many studies. Here, indicate whether each material, system or method listed is relevant to your study. If you are not sure if a list item applies to your research, read the appropriate section before selecting a response.

Materials & experimental systems

n/a	Involved in the study	
☒	☐	Antibodies
☒	☐	Eukaryotic cell lines
☒	☐	Palaeontology and archaeology
☐	☒	Animals and other organisms
☒	☐	Human research participants
☒	☐	Clinical data
☒	☐	Dual use research of concern

Methods

n/a	Involved in the study	
☒	☐	ChIP-seq
☒	☐	Flow cytometry
☒	☐	MRI-based neuroimaging

Animals and other organisms

Policy information about studies involving animals; ARRIVE guidelines recommended for reporting animal research

Laboratory animals	N/A
Wild animals	N/A
Field-collected samples	Samples were collected from a single plant growing in Edinburgh (55.922684 °N, 3.170703 °W) in April 2014. Seeds were germinated in 10-cm round Petri dishes containing distilled water in 16 h light/8 h dark conditions (100µmol m−2 s−1, 25°C during the day, 23°C during the night) for two weeks. They were then transplanted to 7×7×8-cm3 pots with soil/perlite mix 60% v/v Levington's F2+S (Everris), 24% v/v standard perlite (Sinclair), 16% v/v washed horticultural sand 0.3 g l−1 Exemptor (Everris)) and grown in 16 h light/8 h dark conditions in a room with a controlled environment (100µmol m−2 s−1, 21°C) for four weeks. Plants were transplanted into 4-

l pots with peat/sand mix (83% v/v medium peat (Clover), 21% v/v washed horticultural sand, 3 g l−1 garden limestone (Arthur Bowers), 1 g l−1 Osmocote Exact Standard 5–6 months (Everris), 0.4 g l−1 Exemptor (Everris)) and transferred to a glasshouse with ambient light supplemented to ensure a 16-h day (minimum intensity of 250μmol m−2 s−1, 06:00–22:00 GMT) and temperature of 21°C during the day, 18°C during the night.

Ethics oversight	N/A

Note that full information on the approval of the study protocol must also be provided in the manuscript.
